# Green nephrology and eco-dialysis: a position statement by the Italian Society of Nephrology

**DOI:** 10.1007/s40620-020-00734-z

**Published:** 2020-04-15

**Authors:** Giorgina Barbara Piccoli, Adamasco Cupisti, Filippo Aucella, Giuseppe Regolisti, Carlo Lomonte, Martina Ferraresi, D’Alessandro Claudia, Carlo Ferraresi, Roberto Russo, Vincenzo La Milia, Bianca Covella, Luigi Rossi, Antoine Chatrenet, Gianfranca Cabiddu, Giuliano Brunori, On the Behalf of Conservative treatment, Physical activity and Peritoneal dialysis project groups of the Italian Society of Nephrology

**Affiliations:** 1 Nephrology, Centre Hospitalier Le Mans, Le Mans, France; 2 Department of Clinical and Biological Sciences, University of Torino, Turin, Italy; 3 Department of Clinical and Experimental Medicine, University of Pisa, Pisa, Italy; 4 Nephrology and Dialysis Unit, IRCCS “Casa Sollievo Della Sofferenza” Scientific Institute for Research and Health Care, San Giovanni Rotondo, Italy; 5 Department of Internal Medicine, Nephrology and Health Sciences, University of Parma, Parma, Italy; 6 Division of Nephrology, Miulli General Hospital, Acquaviva delle Fonti, Italy; 7 Department of Mechanical and Aerospace, DIMEAS, Politecnico of Torino, Turin, Italy; 8 Nephology Unit. Azienda Ospedaliera Universitaria Policlinico, Bari, Italy; 9 Nephrology Unit. Hospital “A. Manzoni”, Lecco, Italy; 10 Nephrology Unit. Hospital “G. Brotzu”, Cagliari, Italy; 11 Nephrology and Dialysis Unit, Hospital of Trento, Trento, Italy

**Keywords:** Dialysis, Ecology, Waste management, Sustainability, Pollution, Costs

## Abstract

High-technology medicine saves lives and produces waste; this is the case of dialysis. The increasing amounts of waste products can be biologically dangerous in different ways: some represent a direct infectious or toxic danger for other living creatures (potentially contaminated or hazardous waste), while others are harmful for the planet (plastic and non-recycled waste). With the aim of increasing awareness, proposing joint actions and coordinating industrial and social interactions, the Italian Society of Nephrology is presenting this position statement on ways in which the environmental impact of caring for patients with kidney diseases can be reduced. Due to the particular relevance in waste management of dialysis, which produces up to 2 kg of potentially contaminated waste per session and about the same weight of potentially recyclable materials, together with technological waste (dialysis machines), and involves high water and electricity consumption, the position statement mainly focuses on dialysis management, identifying ten first affordable actions: (1) reducing the burden of dialysis (whenever possible adopting an intent to delay strategy, with wide use of incremental schedules); (2) limiting drugs and favouring “natural” medicine focussing on lifestyle and diet; (3) encouraging the reuse of “household” hospital material; (4) recycling paper and glass; (5) recycling non-contaminated plastic; (6) reducing water consumption; (7) reducing energy consumption; (8) introducing environmental-impact criteria in checklists for evaluating dialysis machines and supplies; (9) encouraging well-planned triage of contaminated and non-contaminated materials; (10) demanding planet-friendly approaches in the building of new facilities.

## Introduction: the global context

In what is called his “green encyclical letter”, Pope Francis wrote that reducing pollution and protecting an endangered world were a priority for humankind: “The urgent challenge of protecting our common home includes a concern for bringing the whole human family together to seek sustainable, integral development, for we know that things can change” [1]. The need to preserve our planet, for the sake of all living creatures, was likewise dealt with by the Dalai Lama in a series of talks and was the topic of an interfaith conference attended by exponents of all the major religions [2, 3]. Given that Greta Thunberg was called the icon of the year 2019 by Time magazine, and that the terms pollution, sustainability, carbon footprint, and ecology are increasingly encountered in talks by politicians, the newspapers, hotel rooms, advertisements and on public transport, it appears that time is ripe for applying a “green policy” to all our activities [4].

The issue is not new; the term “throwaway society” was coined in the fifties, at the beginning of the large-scale introduction of electrical appliances in households in developed countries. The Collins Dictionary defines it as “a society full of excessive consumption and waste of food, products, etc.” [6]. The green encyclical letter states: “a throwaway culture (…) quickly reduces things to rubbish” [1].

Finding a way between catastrophic forecasts (we will soon reach the point of no return, or it is already too late), cautious optimism (the process could at least partially be reversed) and revolutionary achievements (technology is associated with a dramatic increase in life expectancy) is not easy, and physicians have not been trained to reflect on the environmental effects of their clinical choices and work habits.

Things are changing and, in spite of the small number of studies regarding the impact of nephrology care on the environment, influential meetings, such as the World Congress of Nephrology in 2019, have recently devoted time to discussions of green nephrology [6].

## Dialysis in the global context

Dialysis is at the heart of the matter as it is one of the most important waste producers in medicine [7–10]. Waste management involves not only ecological factors, it also has an important economic impact, since the disposal of potentially hazardous or contaminated waste, which by definition is all hospital waste that has been in contact with any kind of biological fluid, can be extremely expensive, and represents a problem increasingly felt to be crucial in developing countries [10–15].

Water, energy, and waste management in dialysis account for a relevant proportion of the cost of a session [10].

Shifting from the concept of eco-dialysis to the concept of green nephrology is not only semantic: green nephrology involves re-thinking all daily activities. Furthermore, it involves rethinking dialysis machines with a cradle-to-cradle philosophy, building planet-friendly facilities and choosing renewable energies [16–21].

Expanding the concept of green nephrology to the clinical practice, the best way of dealing with dialysis waste is by avoiding, retarding and reducing the use of dialysis; what is more planet-friendly than a plant-based diet, with a limited consumption of animal derived protein, in particular red meat? [22–24]. Similar considerations apply to the inclusion of programs of physical activity as a part of care, with the potential advantage, among others, of reducing pill burden [25, 26]. The issue of green nephrology, therefore, merges with many others, including prevention, healthy lifestyle, adequate physical exercise and avoidance of processed food, thereby making green nephrology the hub of a comprehensive, individualised approach to problem solving, at the single-patient, social and ecological levels.

It was Professor John Agar, acknowledged as the inventor of the concept of green nephrology and eco-dialysis, who first found constructive and imaginative ways to deal with the difficulties being faced by home dialysis patients treated with intensive haemodialysis, who experienced better health at the price of high expenses for water and electricity, an issue not previously perceived as critical by physicians working in dialysis facilities [9, 16, 19, 27–31]. His engagement resulted in a series of papers from Australia, followed by the establishment of a Green Nephrology Network in the UK within the NHS’s Sustainable Healthcare Programme and by a recent position statement by the Australia New Zealand Society of Nephrology. The Green Nephrology network has produced estimated annual savings to the UK healthcare system of 10 million Euros, as the result of planet-friendly, water and electricity saving initiatives [17, 32]. Along the same lines, the European Renal Association-European Dialysis and Transplant Association has committed to a broad range of initiatives aimed at promoting green nephrology in Europe [33].

Against this background, the Italian Society of Nephrology is proposing a position statement on eco-dialysis and green nephrology.

Due to the scattered evidence available on all these questions, we will not include the usual evidence levels in the recommendations, but will comment on the sources of information and on the quality of the studies.

## Definitions and nomenclature

1. The definition of medical waste is agreed on at the level of the World Health Organisation (official documents available).
2. The laws regulating medical waste management differ in different countries (reference documents and local dispositions available).
3. The costs of waste management are important in the definition of the indirect costs of hospital treatment (scattered data, requiring continuous updating).
4. The “3R” analysis (reduce, reuse, recycle) is a schematic way of analysing the potential for action when seeking to reduce the impact of waste (few examples in nephrology care; examples from different sources available).
5. The waste cycle follows different pathways; these include cradle-to-grave, grave-to-cradle and cradle-to-cradle (few examples in nephrology; examples from different sources available).

A definition of medical waste is available in the Medical Waste Tracking Act of 1988, which broadly defines it as “all waste materials generated at healthcare facilities, such as hospitals, clinics, physician's offices, dental practices, blood banks, and veterinary hospitals/clinics, as well as medical research facilities and laboratories” [34]. The Medical Waste Act specifically refers to dialysis as follows: "Dialysis wastes that were in contact with the blood of patients undergoing hemodialysis, including contaminated disposable equipment and supplies such as tubing, filters, disposable sheets, towels, gloves, aprons, and laboratory coats” [34, 35]. Dialysis waste is divided into contaminated and non-contaminated waste, indicating that the latter (paper, tubing and bags which were not in contact with biological fluids) can be disposed of as normal domestic waste is, unlike contaminated waste, which needs to be processed in prescribed ways [34, 35]. While non-contaminated materials may be reused or recycled, it is generally held that potentially contaminated material should not.

In Italy, waste management is regulated by laws and ministerial decrees, the most important of which were enacted in the new millennium (D.P.R. 254/03 and D. Lgs 152/2006), basically applying the provisions of the Waste Management Act, from the definition of different types of hospital and medical waste to the rules to be followed for transportation and final disposal [36].

The cost of medical waste management is very different across countries; waste may be calculated by volume or by weight, the latter being the most frequent choice in Italy; final disposal is usually by incineration, which may be a significant source of pollutants and is relatively expensive. Legal provisions and definitions are also different, thus adding to uncertainties and making comparisons difficult [37–39].

**Fig. 1:**
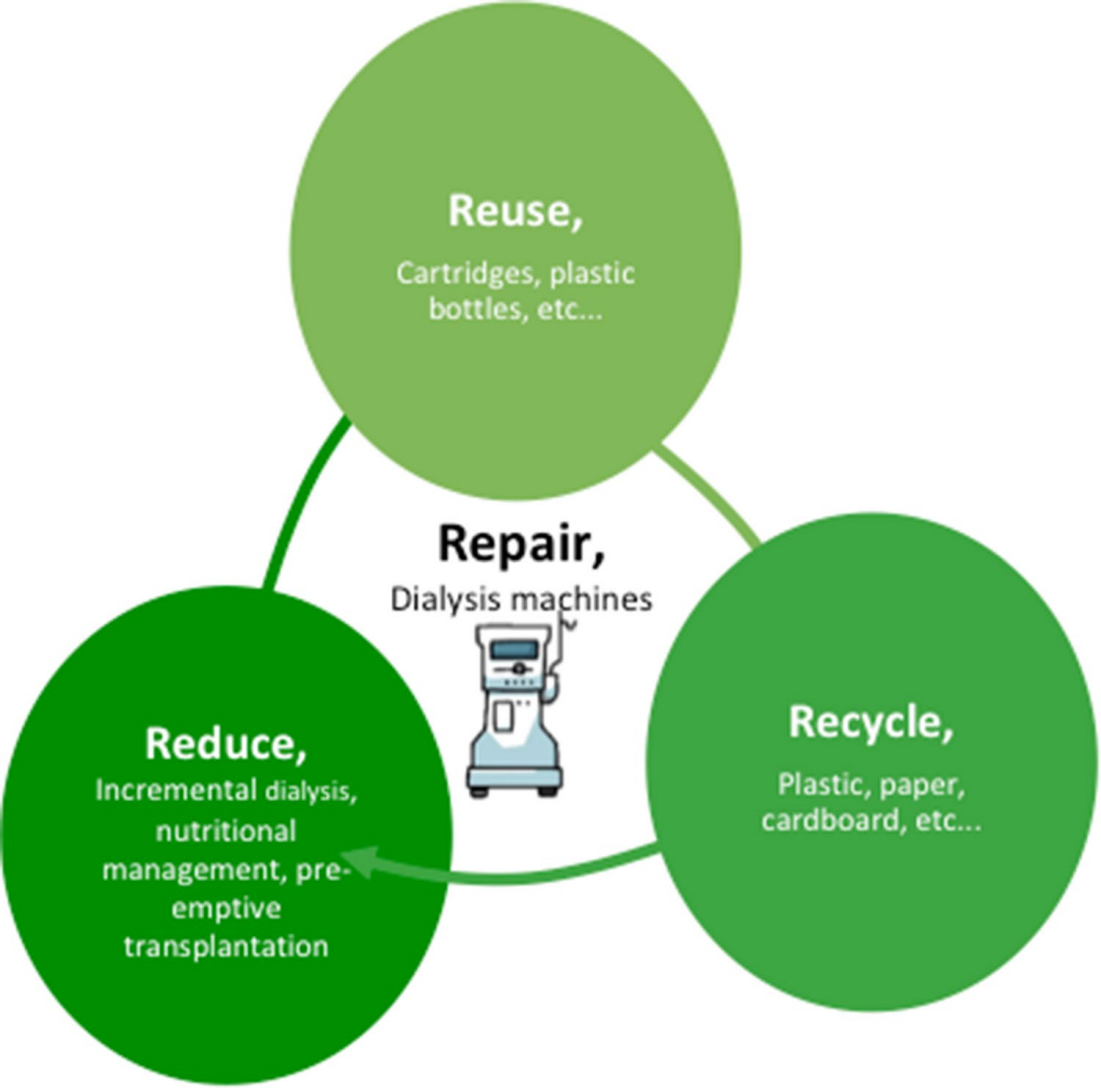
The “R” cycle, and its potential application to dialysis treatment

Awareness of a 3R-approach (reduce, reuse, recycle) is rising, but there are still relatively few virtuous examples, especially in nephrology [40–43]. A fourth R, repair, has recently been added, an indication of growing awareness of the importance of moving away from a throw-away mentality (Fig. 1) [5].

Reduction of the quantity of waste products is the first issue and could be applied at all levels: reducing the number of patients starting dialysis by the wise use of conservative and nutritional treatment; reducing the number of dialysis sessions by employing incremental dialysis strategies; reducing water waste, by tailoring dialysate flow to specific needs; reducing contaminated waste by careful triage of dialysis waste products. Further elements, such as reducing the impact of packaging, shortening lines or optimizing some dialysis components, could become possible through cooperation between physicians and manufacturers.

Reuse in nephrology is often perceived with a negative connotation, calling to mind the controversial practice reuse of dialysers (cfr waste management). However, there is great potential for reusing some dialysis disposables that do not come into contact with blood, such as bicarbonate cartridges.

Recycling is another question that is often overlooked, Dialysis is a great producer of plastic waste and at least a part of non-contaminated plastic items could be recycled, but in the absence of specific programs, this rarely occurs and often falls beyond the control of dialysis units. Furthermore, hospital programs of systematic recycling of items that could (and should) be recycled in the household, such as paper, glass, food, non-medical plastic items, are, at best, non-systematic.

Repair is a further issue that is in sharp contrast with the present attitude to in toto discarding of devices and supplies, including dialysis machines, significantly contributing to the electronic waste (e-waste) produced by dialysis. Repair is not only important for prolonging the life of equipment, but also because it can serve as the basis of a more comprehensive approach to life-cycle analysis and cradle-to-cradle technology, described below (Fig. 1).

Life-cycle analysis is a more comprehensive way to study the ecological impact of a piece of equipment. The starting point is either the beginning (cradle) or the end of the process (grave). In general, the study starts from the best-known part of the lifecycle: the cradle for the industry and the grave for the final users. Reusing or recycling the whole object or its parts may be included in the cycle; however, the recycling process is often a down-cycling one, which, by prolonging the life of the object or its components, does not stop it from being reduced to rubbish. This process is summarised in the linear economy that characterises the “take-make-waste” cycle.

**Fig. 2:**
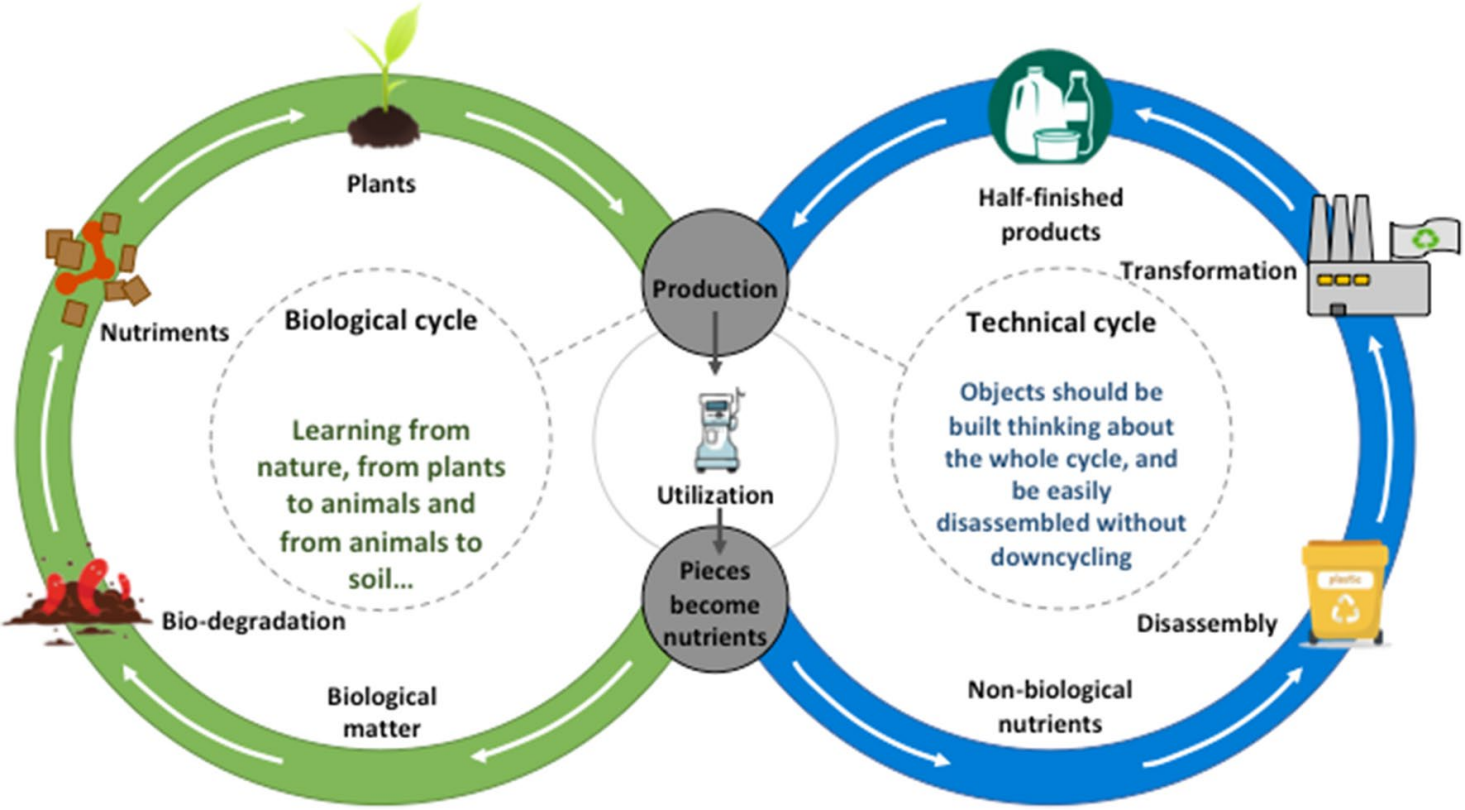
The circular “cradle to cradle” model: technical waste should nourish the technical cycle, similar to organic waste, nourishing plants and being continously recycled in the biosphere

Nature functions with another logic. The law of the conservation of mass, “nothing is lost, nothing is created, everything is transformed”, attributed to the chemist Antoine Lavoisier, recalls the theory that “in everything there is a share of everything” propounded by the 5th-century B.C.E philosopher Anaxagoras, often cited as the basis of the concept of a circular economy, which holds that a different approach is feasible and can benefit both the planet and the end-users [44–47] (Fig. 2).

Along the same lines we find the term biomimicry (or biomimetics), an approach to solving problems based on natural processes, and the cradle-to-cradle approach [48–51]. The idea can be summarised by the example of a dead tree whose organic wastes nourish new plants, without losing their biological components. The premise of the cradle-to-cradle design is that the technological material we use should be rethought starting from easily disassembled components which can be reused, recycled or restyled into equivalent ones, thus nourishing a new generation of objects. For example, it should be possible to disassemble a dialysis machine into its basic components, such as plastic, metals etc. and each of them could then be reused, reassembled or recycled without losing its original “value” [10, 20].

## Outline

### Issues and themes of the potential impact of green nephrology

The present position statement deals with main two topics: technology and clinical practice. In our discussion of the former question, we have sought to identify feasible interventions that would make it possible to reduce the dialysis carbon footprint, a comprehensive term encompassing all types of energy expenditures, ultimately including waste management. In dealing with the latter, we have focused on how a green nephrology approach highlights the importance of those aspects of care that are too often considered ancillary, such as diet or physical activity, also as a means of reducing drug burden and improving survival.

The discussion of technology includes the following four issues: water conservation; energy conservation; waste management; industrial design.

The discussion of clinical issues focuses on three topics, reflecting our view that green nephrology needs to become a comprehensive clinical approach: nutritional management; physical activity; choice of renal replacement therapy.

## Technology

### Water conservation

1. Reverse osmosis for haemodialysis should do as much as possible to recirculate rejected water (many studies available: modest, if any economic gain, relevant ecologic gain).
2. Water ultimately rejected by reverse osmosis should be reused for different purposes (few studies available: probable economic gain, relevant ecologic gain).
3. Water consumption should be considered in tailoring dialysate flow (see personalisation of dialysis).
4. Simple planet-friendly household practices should be implemented in all dialysis facilities and the importance of these measures should be explained to healthcare professionals and patients (few reports of water-friendly choices; common-sense statement, however not in place in the vast majority of hospitals).
5. Studies are needed to assess the amount of water actually needed for PD treatment, the economic advantages of reusing water from reverse osmosis, and the use of rejected dialysis water for agricultural purposes.

Haemodialysis and haemodiafiltration (HDF) consume a relatively large quantity of water, a natural resource unevenly available worldwide. Water consumption in dialysis depends on three major factors: the way the water is discharged from reverse osmosis, and type of reverse osmosis; the way the dialysate and reinfusate are prepared; the prescription of the dialysis session.

Standard reverse osmosis discharges more water than it actually produces for dialysis; the recovery ratio, i.e. the percentage of depurated water used for dialysis, can be over 80%, if the newer systems are employed; these are more expensive and the lifespan of the reverse osmosis membranes is often considerably shorter, especially when reverse osmosis is not coupled with a water softener system [9, 19, 27, 31, 52–57].

To produce the dialysate (120 L per session for a dialysis session of 4 h with a standard dialysate flow of 500 mL/min) the total water consumption with a standard reverse osmosis can be over 300 L and, according to an Australian study, when pre-treatment priming, rinsing and sterilisation of the system are added in, a single haemodialysis session can consume up to 500 L of water [31, 58].

Centralised water preparation systems, available since the late 1970s, and particularly diffused in Japan, are increasingly being used: they combine water treatment with dialysis fluid preparation, both for haemodialysis and for haemodiafiltration [53–57]. This partly compensates for the higher costs of HDF and the avoidance of plastic bags for reinfusion is of obvious interest not only for reducing waste, but also as for water consumption. Producing 1 kg of plastic can require up to 180 L of water. While the water footprint depends on the type of plastic, an empty 2-L dialysate bag weighs about 150 g, whether it is used in PD or HDF, thus consistently adding to water consumption [31, 59].

The centralised water preparation system may simultaneously supply acid concentrate (solution A) to up to 50 patients. Proportioning is computer-assisted, using either fixed proportioning or ‘servo-control’ based on serial measurements of conductivity. The basic concentrate (solution B) is combined at each patient’s monitor. Powder concentrate is generally preferred because it is easier to manage and requires less storage space, which reduces shipping costs [53]. However, a central proportioning machine should have a redundant control system including multiple conductivity monitors, central processing units, power supply units, flow meters and proportioning pumps for emergency backup.

Independently from its quantity, the reject water produced by reverse osmosis is purified tap water that has already passed through a depuration cycle, during which particulate matter, chlorine, chloramines and other potentially harmful substances are removed; this water is commonly considered unfit for human intake due to lack of chlorine [58]. As brilliantly demonstrated by our Australian colleagues, the reject water never contacts the dialyser or the patient and bears no more infectious risk (in fact far less) than tap water. Apart from a mild increase in conductivity, the quality of this water falls within the limits set by the World Health Organisation for potable water [31, 58]. The inventive Australian team found several ways to reuse this reject water. In their earliest experiments, rather than letting it go down the drain, they redirected the water into a storage tank to serve other areas of need (the hospital’s central sterilising department for steam generation, toilets, janitors’ stations and gardens) [60]. The same principle was applied to home haemodialysis, where clean reject water was used in laundries, toilets and gardens, to at least partially offset increased water costs for home dialysis that patients were not reimbursed for [31, 61].

To give an idea of the potential savings that ensued, one dialysis service in the UK has reported savings of up to 4 million litres of water per year with a new ergonomic water system [32]. UK, Australian and French data show that adding water-conserving devices to an existing reverse osmosis system can be water saving, as well as economically sound [58, 61–64].

We were able to find only one study on the use of reject dialysis water in agriculture. Done in Morocco, it investigated the possibility of using “contaminated water” drained from dialysis machines for agricultural purposes. Of note, this discharged water showed levels of organic matter and bacterial counts that fell within the limits set by the World Health Organisation and the United Nations Food and Agriculture Organisation for agricultural purposes [65]. Indeed, on this same line, we may mention the current research about urine recycling, especially in space agencies, whose results could be translated to the employment of the dialysate for different purposes.

Whatever we do, haemodialysis is a water-hungry treatment. Conventional thrice-weekly 4-h haemodialysis, with a dialysate flow of 500 ml/min, and a standard osmosis, consumes about 20,000 L of water per year. In the context of a centralised preparation system, the water demands of haemodiafiltration are 10–30% higher, depending on the exchange volumes employed. No figures are available in the case of use of industrial bags for hemodiafiltration or, as later discussed, for peritoneal dialysis.

In the late nineties, at a time when the focus was on increasing dialysis efficiency for all patients, some centres increased dialysate flow to up to 700–800 ml/min, a policy which is now being reconsidered. The modest gain in efficiency (usually estimated as less than 20%) is in fact attainable only in patients with concomitant high blood flow, and even in such cases the clinical advantage may be dubious [66–71]. At present the balance may point towards a lower dialysate flow in elderly patients with low metabolic needs, where optimal dialysis is probably a compromise between depuration and depletion [72, 73].

Some of the new small home haemodialysis machines recirculate low-flow dialysate, with the idea that a favourable solute gradient can be attained at much lower flow rates throughout the haemodialysis session, in particular on short daily haemodialysis [74]. These experiences also suggest that, at least in elderly patients with low metabolic needs, poorly functioning vascular accesses and poor nutritional status, lowering the dialysate flow can be attained without having negative consequences (and possibly producing some positive ones) for the patient.

At first sight, in terms of water consumption, peritoneal dialysis (PD) has a much more planet-friendly profile than haemodialysis. Depending on dialysis prescription, PD uses 6 to 12 L per day of dialysate. However, dialysate comes packaged in plastic, which, according to the Australian data, has a large water footprint, an average of 25–30 L per plastic bag. We lack precise data on how much water is needed for PD fluid production as manufacturers, protected by laws on industrial secrecy, are not obliged to divulge this information [31]. If we consider that an average CAPD patient on four 2-L exchanges a day, and consider that each dialysate bag produces a water footprint of 25 L, assuming that preparation of ultrapure dialysis fluid involves a 50% water discharge, we end up with about 120 L of water per day—840 L per week—which is roughly similar to the 240 L of water per dialysis session (with a reject rate of 50%), producing 720 L per week not considering rinsing and sterilisation [31].

While several groups are presently working on systems able to produce PD fluids at centre or home level, no such system is presently available on the market. The most advanced one seems to be the Ellen Medical Affordable Dialysis System, presented at the annual meeting of the American Society of Nephrology in 2019, which encompasses most of the important green features: minimal water wastage, solar power, portability [75].

Big issues, such as dialysis prescriptions and new machines and devices, should not distract attention from simple, feasible, goals: in the hospital, like at home, simple measures, such as dual flush toilets, flow restrictors on taps, water collection from roof drains, should be integrated not only to reduce the hospital’s carbon footprint, but also as a means of teaching people to use water wisely [31, 76, 77]. The issue of new facilities will be discussed below.

### Energy conservation

1. As far as possible, renewable energy should be chosen to supply power in haemodialysis facilities (few studies available: modest, if any economic gain, potential ecologic gain).
2. Simple planet-friendly household practices should be implemented in all dialysis facilities, where they also serve as a means of making patients and healthcare professionals aware of the issue (few reports on planet-friendly choices; common sense statement, however not in place in the vast majority of hospitals).
3. Including the issues regarding energy preservation and sustainability in the educational programs for patients and staff may help spreading the culture of environment preservation (no study available; in theory a powerful way to spread consciousness).

Differently from the many actions that can be proposed to reduce the impact of thirsty dialysis, no specific action has so far been identified to reduce the “hungry dialysis” demand for electricity.

As usual, our Australian colleagues have provided important, and so far unique studies: they calculated that a dialysis session (performed with a Fresenius haemodialysis machine with individual Gambro water treatment) consumed on the average 6.2 kWh per 4–5 h session plus set-up and post treatment disinfection [78]. In two satellite units equipped with a central reverse-osmosis, power consumption per session ranged between 12.0 and 19.6 kWh, the difference being mainly due to the energy demand of the water treatment system [31]. According to the Australian data, this means that the yearly individual “power need” is almost doubled in dialysis patients [19, 31, 78].

While there is little hope of changing this situation without cooperation from manufacturers, a goal that is within our reach is systematically choosing renewable energy to supply power supply in dialysis centres or at home. While the source of energy (water, wind or sun) will vary according to setting, a study from sunny Australia suggests that solar power can be efficiently used both in dialysis units and in patients’ homes. The time needed to obtain a return on investment was initially estimated at 7–8 years [31, 78]. However, since the first publication of the study, costs have almost halved, and in the near future we can look forward to relevant savings with the use of solar power. These savings will probably be confirmed even when considering the carbon footprint produced by solar devices.

Adding specific meters after the water treatment system or for electricity may allow quantifying what is related to dialysis and what is related to the other activities of the hospital or of the clinic, as well as measuring the effect of action plans [79].

While even less is known about the energy requirements of peritoneal dialysis, it should be kept in mind that this is the only type of dialysis that can be proposed, albeit with enormous difficulties, in extreme conditions such as in settings without electricity [80, 81]. Studies are needed to assess power consumption on PD, including water treatment, cycler assisted PD, and transportation.

Once more, we should consider that the hospital is at least as important as our home, and once more simple measures, such as choosing eco-friendly lighting and heating (and cooling) systems and automatic power shutdown of electrical equipment, should become an integral part of patients’ and caregivers’ daily lives [31]. In such a context, including sustainability among the issues that are discussed in the educational programs for dialysis patients may be a precious tool to enhance understanding and reinforce behaviours.

### Waste management in dialysis

1. Triage of contaminated and non-contaminated waste should be the core procedure of dialysis waste management (common sense evidence).
2. Recyclable waste, including paper and plastic from packaging, should be transferred to appropriate settings (limited availability of facilities actually proposing systematic recycling).
3. Reusable items (tourniquets, bicarbonate cartridges, etc.) should be personalised and reused (no studies, but large differences on the global dialysis scale)
4. Non-recyclable, potentially contaminated waste can undergo high temperature transformation (limited, positive experience)
5. Home dialysis waste management will need to be adapted to local regulations (limited studies, most from Australia).

Dialysis, in all its forms, produces a relevant quantity of waste, be it per session (extracorporeal dialysis), or per day (peritoneal dialysis). All materials that come into contact with body fluids are considered potentially contaminated and have to be disposed of separately. It has been calculated that one dialysis session produces between 1.5 and 8 kg of waste, according to the attention paid to waste disposal, in particular to carefully emptying dialysis disposables [19]. Contaminated, or hazardous waste, according to different regulations, must be incinerated or sterilised, before final disposal, usually in landfill. The cost of its management is high (in Italy from 2 to 7 Euros per kg) [19, 31]. As a consequence, the cost of indiscriminate waste disposal after a dialysis session (disposing all supplies together, without attention to careful emptying) may be as high as 50–70% of new single use disposables [19]. General (household-type) waste is usually directly disposed of in landfill or incinerated; plastic-derived toxins can leak into the soil and groundwater, while organic waste can be a source of methane [19, 31, 82–84].

Waste management starts from the materials used in packaging, which is often relatively large and made of plastic and paper. The key to reuse is the appropriate triage of the different items and the avoidance of “contamination”, i.e. mixing potentially recyclable materials of different types. This is true for cardboard and paper, for example, and is the major problem encountered in disposing of different types of plastic items, as the recycling procedure generally requires “pure” material, i.e. one type of plastic only, without glues, labels, and non-plastic elements.

After the dialysis session, “wise” differentiation includes the careful emptying of fluids trapped into the dialysis supplies. Differentiation is not necessarily time-consuming, but it does require attention. According to an Italian study, a trained nurse can complete the procedure in about one minute for bicarbonate dialysis and in two to three minutes for off-line HDF [19].

In the absence of guidelines, training in correct waste management procedures is critical in minimizing environmental impact. Separation should take place as close as possible to where the waste is generated [7, 30, 85–87].

Well-separated, non-contaminated, “clean” material is however not synonymous with recyclable material. There is limited compatibility between different plastics in terms of reuse and, according a previous experience in Italy, less than 30% of the separated materials from a dialysis session are actually recyclable, and less than 10% actually undergo the process [19].

A high percentage of plastic waste is made of polyvinyl chloride (PVC); PVC is potentially recyclable, but when PVC is mixed with other plastic materials this usually precludes recycling [30, 31]. Furthermore, when PVC is discharged in landfill or incinerated, it can release highly toxic dioxins and chlorinated organic compounds into the atmosphere and in the soil [31, 88, 89].

Some studies in non-medical settings suggest that several types of plastic may be successfully recycled into concrete [90, 91]. On this line, one alternative that has been recently proposed for contaminated plastic deriving from the dialysis waste is to use steam sterilization, followed by shredding into confetti-sized pieces; these are then incorporated into concrete [92, 93].

There is a need for a close collaboration between hospital services, the dialysis wards and the local facilities to maximize the impact of careful triage and improve the recycling of dialysis materials, a policy could serve as a good example to be adopted in other hospital departments.

There is an almost complete lack of data on the quantity and quality of waste arising from peritoneal dialysis. The only study we were able to find is from the UK, and reports that 4 daytime exchanges in PD generate about 1.7 kg of solid waste per day; more than half of this is made of polyvinyl chloride [7]. According to the authors, the yearly amount of plastic waste generated by PD was higher than from haemodialysis (617 kg versus 390 kg). We were not able to find data on waste production in automated PD.

The issue of the reuse of dialysers has not been developed in this discussion, because the practice has been banned in Italy, as well as in most European countries, for over 20 years. Notwithstanding the increased risk of infection that is often reported, reuse is a highly complex issue, probably also because most of the measures of “biocompatibility” are indirect (like pre-dialysis beta2 microglobulin level) and, as it is common in the field of dialysis, it is very difficult to disentangle the effect on mortality from that of the overall patient care, of which reuse may be a reflection [94, 95]. The low quality of the evidence in the available studies, acknowledged in the only systematic review we were able to retrieve, and the heterogeneity of the reuse modalities suggest that the time may be ripe for re-thinking this practice in the different context of ecological sustainability. In this regard, future studies should balance the advantages in reducing plastic waste with the need for using potentially toxic chemicals for membrane sterilization [94–97].

### Projecting a new dialysis unit

1. A planet-friendly hospital facility employs solar power, renewable energy, natural materials and materials with a negative CO_2_ balance whenever available; plastic and synthetic materials are avoided (common sense, interesting experiments, particularly in Australia).
2. Reusable, natural materials should be preferred whenever possible in daily activities in hospitals. The advantage of using, for example, cotton sheets, and lab coats, needs to be weighed against the carbon footprint produced by washing, sterilisation and transportation (common sense, interesting experiments, in particular in Australia).
3. Healthy diets, consisting of foods that have been tested to ensure they are free from pesticides, contaminants and chemical preservatives, should be prescribed. As far as possible, fresh, locally grown produce should be preferred (common sense, toxicity well known in CKD patients).

If the health of human beings is also a reflection of the health of the environment in which they live, hospitals should be examples of a wise, conscious use of natural resources.

This should start from employing eco-friendly materials when facilities are built. The idea of hospitals as paladins of a green approach to medicine is appealing, and leaves space for many kinds of initiatives, such as recycling all suitable materials, reducing single-use synthetic consumables that can safely be replaced by reusable natural ones (e.g. bedding and lab coats), and utilising natural materials whenever possible. Similar suggestions have emerged from laboratory experiments [31, 40–42].

Things are never simple: while in the past many hospitals worked as self-sufficient autarchic structures, providing a vast array of services, including laundry rooms, sterilizing facilities and kitchens, now, in many settings, there is a tendency to outsource these activities, choosing to cut expenses instead of improving efficiency. This questionable, albeit understandable choice has the side effect of adding the ecologic costs (carbon footprint) of transportation to working areas (e.g. laundries), or reliance on processed food, which often entails increased use of plastic packaging, a practice that from a green perspective should not be encouraged.

For similar reasons healthy, local, natural food should be preferred and all precooked and processed foods should be avoided, with the double advantage of contributing to patients’ health, and exploiting hospitalisation as a valuable occasion for nutritional education [98–100].

No hospital in Italy meets these standards, and this is also in other European countries. Nephrologists are not trained in these matters and the time they have to dedicate to green issues is frequently limited by an overwhelming work schedule. However, since nephrologists usually spend more time in the hospital than at home, this should lead them to reconsider the importance of a healthy hospital environment not only for patients but also for the healthcare team [101, 102]. The importance of a friendly, healthy, and when possible colourful and stimulating hospital setting has been demonstrated in geriatric medicine, paediatrics and psychiatric wards. While examples of carefully designed paediatric dialysis wards are available, there is less experience with the adult (or geriatric) population [103–105]. In this broader sense, attention to a green environment should merge with attention to a healthier, friendly setting of care.

### Relationship with the health-care system and with the industry

1. The hospital management should be involved as much as possible in all the “green” initiatives, to allow optimizing the results; economic evaluations may play a role in this engagement (once more… just common sense).
2. A close relationship between healthcare professionals and manufacturers is fundamental for favouring the development of devices and machines that answer clinical needs but have a reduced ecologic impact (once more… just common sense).
3. Including specific questions on the sustainability of the methods of production, distribution and waste management involved in the manufacture of supplies and machines, particularly those used in dialysis, can be a way to induce the industry to develop planet-friendly policies (once more… just common sense).

Dialysis a demanding and complex activity, needing infrastructures, medical and nursing staff, a complex organisation, especially in large units.

The hospital management should be involved as much as possible in the “green” initiatives, to allow optimizing the results. Economic evaluations may play an important role in engaging the hospital management or the health care authorities in investing in planet friendly activities.

Dialysis is not only patient care, it requires a close links with manufacturers and providing treatment without restrictions represents one of the highest costs in the healthcare system. The development of dialysis and its shift from pioneer procedure to the industrial era has deeply changed the relationship between nephrology and manufacturers, and nephrologists are now only occasionally (but usually successfully) involved in projects of dialysis machines [106].

Rethinking dialysis from a new viewpoint and adopting new cradle-to-cradle models in industrial design will require reinvesting in shared experiences. As we wait for this to come about, the authors of this statement consider that the systematic adoption of a checklist for comparing information on the different steps involved in the production of disposables and machines would be a feasible way to raise awareness and stress the need for cooperation. The list could include:

- data on packaging (paper, plastic, printed matter, labels, production sites),
- detailed information on the constituents of non-contaminated materials (recyclable, non-recyclable, partially recyclable);
- specifics on the origins of the components in dialysis machines, and on recyclability and reusability of all or part of the materials,
- questions on trace materials in disposables (not declared if in small quantities);
- support from the industry in identifying recycling facilities for disposables and machines.

While it is probably too early to introduce procedures for evaluating each of these items, we suggest considering that planet-friendly transparency, reflected by responses to the questions on the list, should be considered as ancillary quality criteria in selection procedures.

## Clinical themes

### Nutritional and conservative management

1. Clinical programs supporting safe prolongation of the pre-renal replacement therapy (pre-RRT) phase do not only represent favourable clinical and economic investments, but are also planet friendly (sound evidence on clinical and economic issues; no study reporting on the ecologic impact of spared dialysis years).
2. Nutritional management should consider not only the quality of macronutrients, but also ecologic impacts, and should focus on additives, chemical preservatives, pesticides and contaminants (sound evidence on clinical issues; limited evidence on CKD progression).
3. In a wider perspective, healthy nutrition can be considered the first step of a green nephrology approach, in all CKD stages (opinion-based statement, lack of studies on the topic).

Dialysis saves lives, but cannot be considered a planet-friendly treatment.

In this regard, nutritional management is in keeping with the “R” of “Reduction” of the ecologic impact linked to dialysis need, with all the implications previously described [107, 108]. Retarding dialysis has a comprehensive impact on other issues, such as transportation and use of hospital structures, usually lower in pre-dialysis patients than in those on dialysis [109–111].

While the impact of drugs on the overall carbon footprint remains to be established, and is probably higher than previously thought, dietary management merges the advantages of non-pharmacologic treatment with those of reducing uremic intoxication. While acknowledging the well-known caveats of protein restriction, the wise use of low-protein diets, combined with a comprehensive nutritional approach, not only reduces or delays the need for dialysis start, but also favours the use of incremental dialysis schedules [107, 108, 112, 113].

Nutritional management of CKD is a complex issue, appreciation of which goes well beyond the scope of this consensus statement. Nutritional management starts from healthy eating habits and is not limited to reducing “toxic” precursors, such as proteins, phosphate or salt: a low-protein diet is not a cafeteria diet or a junk food diet, even if these diets may have a low-protein content [114–116]. Conversely, reduction of meat consumption, in particular red meat, can have a favourable ecologic impact, given the large carbon footprint of raising livestock [117].

While there is limited evidence of the effect of trace elements, pesticides and chemical preservatives on kidney health, a planet-friendly approach to nutritional issues should consider not only increasing the quantity of plant-based food, but also controlling its quality, avoiding processed food and favouring seasonal products [22, 117–120]. In this regard, nutritional management can be considered the basis for comprehensive management of CKD patients [121].

### Choice of renal replacement therapy has an ecologic impact

1. Implementing kidney transplant programs reduces the need for dialysis, is cost effective and probably also planet friendly (sound evidence on clinical and economic issues; no study reporting on the ecologic impact of transplantation vs dialysis).
2. Home hemodialysis and peritoneal dialysis make it possible to reduce the burden of in-hospital treatments and transportation. PD may reduce the plastic burden. The role of recycling needs to be assessed (limited evidence on waste management in PD patients; potential for recycling unexplored).
3. Incremental haemodialysis can enable us to safely spare dialysis sessions, with similar mortality and lower morbidity (sound clinical evidence; no data on the ecologic effect of sparing dialysis sessions).

Haemodialysis is probably the most demanding renal replacement technique in terms of needs for transportation, energy and water, while waste management may not be significantly different from PD [31]. The carbon footprint of a treatment goes beyond these issues, and includes others such as drug treatment, whose environmental impact is very difficult to evaluate, but may be as high as that of dialysis disposables [31, 122]. Data comparing haemodialysis and peritoneal dialysis is lacking and fail to indicate the true potential for recycling PD materials, which, like urine bags, should probably be classified as urban waste. One obvious advantage of PD is that it reduces the need for patient transportation. Furthermore, preserving residual kidney function, usually easier on PD, is associated with a lower dialysis dose [31, 123–125].

A Chinese study on PD found that about 80% of its carbon footprint was linked to plastic dialysate bags, their outer packaging and cardboard boxes. However, the study, in which the patients were on maximal PD doses, did not consider the carbon footprint produced by delivering drugs and PD supplies [126].

Likewise, home haemodialysis may have a favourable footprint profile, allowing reduction of patient transportation, an advantage that remains evident even when delivery of drugs, supplies and eventually, in the countries where this is required, retrieving dialysis waste, are accounted for [127, 128]; however, home haemodialysis is often synonymous of more frequent dialysis; studies on these issue are highly needed.

The clinical advantages of kidney transplantation are evident and the concept of cadaver organ donation is fully consonant with a cradle-to-cradle approach to resources. No study, to the best of our knowledge, has quantified the effect of kidney transplantation versus dialysis in ecologic terms [31].

Incremental dialysis is of particular interest; the concept, which originated from PD, supports implementing a progressively higher dialysis dose with a progressively reduced residual kidney function [129–135]. Several schedules are available, whose is beyond the aim of this paper. In any case these patient-friendly approaches seem to allow preserving kidney function and lowering dialysis related comorbidity, in particular in association with dietary management [129–135]. From an ecologic point of view, incremental dialysis allows us to limit the carbon footprint, wastes and social costs, although, almost paradoxically, the treatment costs borne by the institutions providing care may be higher [136].

### Physical activity

Physical activity is a crucial component of health and nutritional status and should be preserved in individuals affected by CKD. Physical activity is a natural, planet-friendly treatment, which can make it possible to reduce pill burden while improving the quality of life (sound evidence on clinical and economic issues; no study reporting on the ecologic impact of reducing pill burden).

In a wider perspective, physical activity could be considered the second most important element of a green nephrology approach in all CKD stages (opinion-based statement; lack of studies on this topic).

Patients with CKD or on dialysis often have low levels of both objective and self-reported physical activity, even compared with elderly people in the general population [137]. A sedentary lifestyle is associated with frailty and disability, and reduced physical activity and impaired mobility predict cardiovascular morbidity and all-cause mortality on haemodialysis [138, 139]. There are multiple links between comorbidities, inactivity, skeletal muscle atrophy and dysfunction, fatigue and pain, blood pressure control, depression and lack of motivation [140]. Several barriers, however, retard the systematic implementation of exercise programs dedicated to CKD patients [140–142]. Of note, anti-hypertensives, painkillers and antidepressants are among the most widely used drugs in CKD patients [143–145]. The possibility of reducing the pill burden using a natural approach is not only clinically sound (reducing adverse effects and improving compliance) but can have a relevant impact on the carbon, water and energy footprints produced by drugs [146].

A number of studies have addressed the effects of physical exercise on dialysis efficiency and phosphate clearance. While the data are still inconclusive, the lack of side effects should be underlined [147–151]. Furthermore, while there is still no clear-cut evidence that exercise reduces the incidence of hard cardiovascular outcomes, a positive impact on frailty could reduce hospitalization and associated costs [152]. Exercise can decrease vascular stiffness and improve blood pressure control, so that, at least in some patients, the pill burden can be reduced. More recently, a meta-analysis suggested that exercise retards CKD progression [153].

In summary, while exercise cannot be seen as a panacea, the implementation of this patient-friendly and planet-friendly approach in the care of CKD patients is likely to have a favourable impact not only on quality of life, but also on various aspects of care (such as pain, nutrition, depression), reducing costs and drug footprints.

## From the world to nephrology and back

Our relationship with the environment is not one-way. Changes in the environment, global warming, pollution and the use of toxic substances in agriculture have potential effects on population health and on the development of diseases, including nephropathies.

Climate change is characterized by a ubiquitous increase in average temperatures, quantifiable in about 0.8 °C in the last 50 years, accompanied by increased humidity. It is associated with increases in heatwaves, and the frequency of extreme weather events such as windstorms, floods and droughts. This has a significant impact on morbidity and mortality, in humans and animals [154, 155]. Heatwaves are differently and non-univocally defined; common definitions include an increase of at least 5 °C over the mean temperature for 5 days or longer, or three or more days of unusually high maximum and minimum temperatures in any area [156–158]. Regardless of the chosen definition, their most important impact is on the cardiovascular system and kidney function [158–160]. High temperatures, especially when access to water is limited, can cause multiple kidney hits which may lead to acute kidney injury (AKI), urolithiasis and urinary tract infections [161]. In recent years, epidemics of CKD have been identified in various hot regions of the world. In the early 1990s, an inexplicably high number of deaths from kidney failure described among farmers in El Salvador, led to the concept of a kidney disease of unknown origin (CKDu), not linked to diabetes, hypertension or glomerulonephritis, but rather associated with exposure to high temperatures often in combination with agrochemicals, heavy metals, infectious agents, genetic factors, malnutrition and zoonosis. Similar cases were then reported in India and Sri Lanka, as well in a growing number of Mesoamerican countries [162–165]. While the pathogenesis is not fully clear, heat is probably a relevant catalyst for one or more toxic substances [162–170].

In many of these cases, kidney biopsies showed tubular atrophy, interstitial fibrosis, and glomerulosclerosis, with signs of kidney ischemia, suggesting episodes of prerenal AKI, rhabdomyolysis, tubule-interstitial injury and uric acid crystallization [166–169]. In Western countries, high fructose intake in soft drinks often leads to inflammatory and oxidative tubular damage [170]. Urinary stones and urinary tract infections are increased by dehydration [171]. Furthermore, heatstroke and dehydration can lead to rhabdomyolysis, with hyperuricemia and tubular damage. Acute heat stress may present with a severe clinical picture, with confusion, delirium, liver and kidney failure and electrolyte abnormalities [168]. The evolution of the initial renal involvement is mostly benign, although progression to chronic renal failure is possible [169–174]. In this context, The Lancet is promoting an initiative called “The Lancet Countdown”, bringing together experts to monitor how climate change is affecting health [175].

We live in a polluted world. Kidney function in patients with CKD may be more prone to worsen or patients may develop other types of toxicity when under the challenge of toxic substances.

Diseases like hypertension, obesity and type-2 diabetes are closely associated with an unwise use of resources, and from people’s lack of information. While under-nutrition is still a threat in many countries, over-nutrition of poor quality is a poison for emerging ones and selectively affects the lower socioeconomic strata in high-income countries [176].

### What this position statement did not discuss?

Whenever we consider the “green dialysis-green nephrology” issue in a broader way, we realize how that many important issues we do not touch. Digital pollution is one of them: the dialysis machines are increasingly connected to the web and information on dialysis parameters, medications, labs tests are increasingly centralized. While this sharing system may be a key for better care, its direct and indirect cost in terms of energy consumption has to be established.

It is probably too early for proposing a systematic green approach to the evaluation of new treatment options, like for example xeno-tranplantation, as we lack data on the main present alternatives; such evaluation however, should integrate the clinical practice in the same way as conventional cost issues are integrated in the clinical reasoning, without necessarily affecting the final treatment choice.

Furthermore, nephrologists belong to a professional community that is accustomed to travel all around the world to share data, experience, and research, to increase knowledge and improve patient care. The carbon foot-print of large meetings is surely very high; “flygskam”, the new Swedish concept describing the shame of flying for environmental reasons, may limit this taste for travel, and lead to consider educational alternatives to answer “ecologically” to the needs of training and research.

## Concluding remarks

**Fig. 3:**
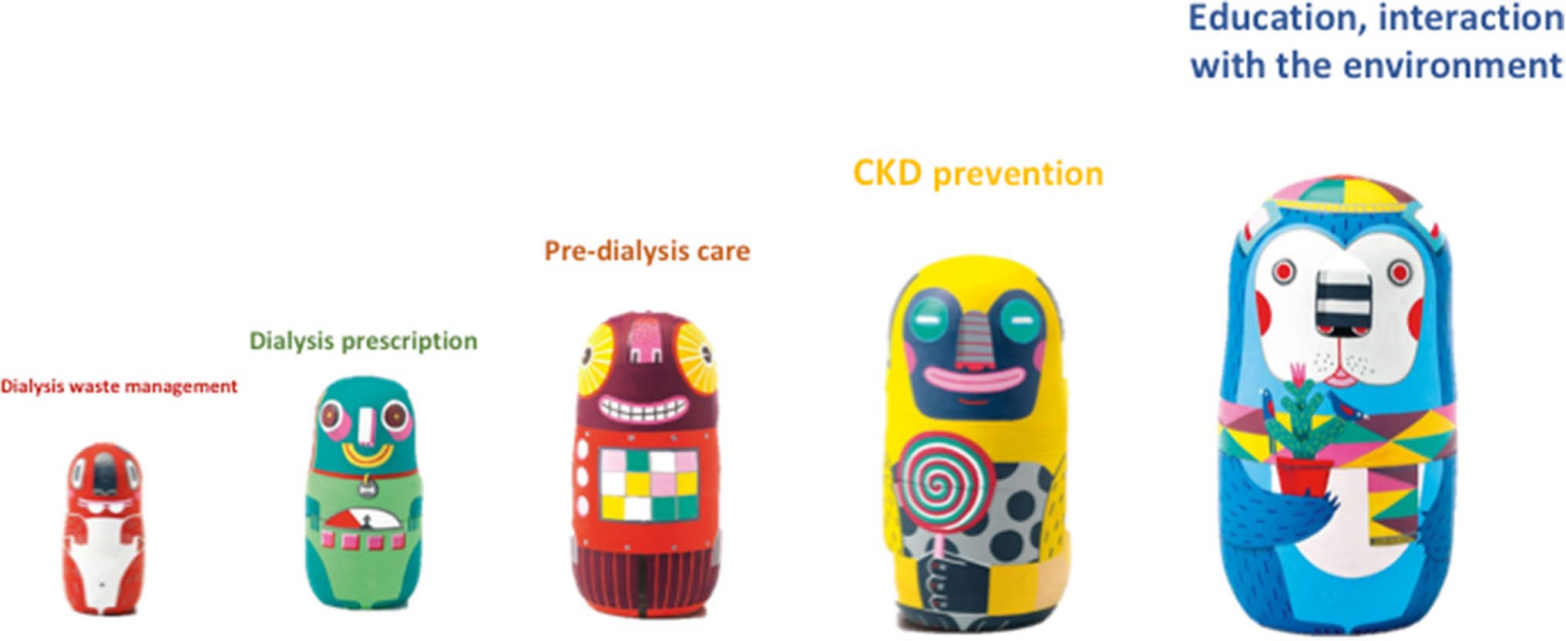
The issues regarding ecology and nephrology are like Russian dolls: the smaller ones, regarding eco-dialysis and waste management, lead to reconsider dialysis prescriptions, predialysis care, and eventually education and prevention of kidney diseases

Unravelling the meaning of green nephrology is like starting from the smallest in a set of nesting Russian dolls, in our case dialysis waste, moving to dialysis prescription, then predialysis care and the prevention of CKD, and finally confronting wider social challenges, including education and social differences (Fig. 3). While overly widening our horizons might be counterproductive, the shift from technical to clinical and from diseases to the world around them underlines the importance of social commitment in nephrology, as well as in all humane professions.

This position statement differs in several ways from other position statements so far produced by members of the Italian Society of Nephrology. First, it is concerned with the relationship between our activities and the world around us, rather that with the management of patients.

Secondly, it is based on limited experience and is not supported by randomised trials or proofs of efficacy, but instead presents indirect evidence from nephrology and dialysis settings.

These limitations are intrinsic both to the novelty and to the nature of the subject discussed and our paper’s limits are shared by the few available position statements on green nephrology, most of which are addressed to increasing awareness of these vital, often neglected issues.

With the aim of combining matters for reflection with practical insights, in keeping with the pragmatic goals of a position statement, we would like to conclude by identifying 10 feasible, affordable actions.

1. Reducing the burden of dialysis (adopting whenever possible an intent to delay strategy, with wide use of incremental schedules).
2. Favouring natural medicine dealing with lifestyle, exercise and diet and limiting drugs.
3. Supporting reuse of household-type hospital material.
4. Recycling paper and glass.
5. Recycling non-contaminated plastic.
6. Reducing water consumption.
7. Reducing energy consumption, and choosing renewable energy.
8. Introducing environmental impact criteria in checklists when evaluating dialysis machines and supplies.
9. Supporting wise triage of contaminated and non-contaminated materials.
10. Demanding planet-friendly approaches in the building of new facilities.
